# Effects of Moist Exposed Burn Therapy and Ointment (MEBT/MEBO) on the autophagy mTOR signalling pathway in diabetic ulcer wounds

**DOI:** 10.1080/13880209.2019.1711430

**Published:** 2020-01-22

**Authors:** Aitian Zheng, Huadan Ma, Xianbin Liu, Qing Huang, Zheng Li, Lin Wang, Na Zeng, Biaoliang Wu

**Affiliations:** Department of Endocrinology, The Affiliated Hospital of Youjiang Medical College for Nationalities, Baise, Guangxi, China

**Keywords:** Diabetes, traditional Chinese medicine, fibroblasts, wound healing rate, PI3K–Akt–mTOR

## Abstract

**Context:**

Burn therapy (MEBT)/moist exposed burn ointment (MEBO) is an effective traditional Chinese medicine method to treat diabetic wound, but the mechanism is unclear. Autophagy has been proved to be closely related with wound healing, so MEBO/MEBT is hypothesized to promote diabetic wound healing by regulating autophagy.

**Objective:**

To explore the mechanism of moist exposed MEBT/MEBO promoting diabetic wound repair.

**Materials and methods:**

Eighty male Wistar rats were randomly assigned to control (*n =* 20) and diabetic group induced by intraperitoneal injection of STZ (*n =* 60), which were further randomly assigned to MEBO, Kangfuxin and model groups (*n =* 20 each). All rats underwent full-thickness skin resection in the back. Wound healing was dynamically observed and wound tissues were collected at five time points for pathological examination, autophagosome and the expression of PI3K, Akt and mTOR.

**Results:**

The healing time in the control group was the shortest, no statistically significant difference was found between the MEBO and the Kangfuxin group (*p =* 0.76). The morphology of autophagosomes ranged large to small, which was the most obvious in the MEBO group. The mRNA and protein expression of PI3K, Akt and mTOR in each group reached the peak on Day 5, the levels in the MEBO group were the highest (*F =* 18.43, 19.97, 15.36, *p <* 0.05). On Day 11, the expression levels in each group began to decline.

**Discussion and conclusions:**

In this study, we discussed the molecular mechanism of MEBT/MEBO promoting the repair of diabetic ulcer wounds through autophagy and PI3K-Akt-mTOR signalling pathway, which provides a new way for drug design in the future.

## Introduction

The incidence of diabetic ulcer wounds has increased year by year. The treatment for diabetic ulcer wounds is a challenging issue (Gupta et al. 2017; Wang and Gao 2018). The Traditional Chinese medicine (TCM) exerts a significant therapeutic effect on chronic ulcer wounds. Moist exposed burn therapy/Moist exposed burn ointment (MEBT/MEBO) is a TCM surgical treatment that greatly improves the effectiveness of ulcer wound healing in clinic (Al-Meshaan et al. 2008). Autophagy is an important biological phenomenon that plays important roles in maintaining cell homeostasis and cell regeneration (Kocaturk et al. 2019). It scavenges damaged organelles and harmful proteins in the cytoplasm, is involved in anti-infection, regulates wound inflammatory response, provides metabolic energy, participates in neovascularization in the wound and epithelial regeneration, maintains intracellular environmental stability and promotes cell regeneration (Mahapatra et al. 2019). Thus, autophagy is very important for repair and healing of ulcer wounds. The occurrence of autophagy is negatively regulated by phosphatidylinositol 3-kinase (PI3K), protein kinase B (Akt) and mammalian target of rapamycin (mTOR) signalling pathways (Li et al. 2019). Many studies proved that MEBT/MEBO could promote ulcer wound healing in diabetic rats, but the mechanism still remains unclear (Jie Hui et al. 2016; Tang et al. 2017). Therefore, this study observes the influence of MEBT/MEBO on PI3K–Akt–mTOR pathway and autophagy so as to preliminarily explore the repair mechanism in diabetic ulcer wounds.

## Materials and methods

### Experimental animals

Eighty healthy male Wistar rats weighing 230–260 g were provided by the Experimental Animal Centre of Youjiang Medical College For Nationalities [License number: SCXK Gui 2017-0003 (Manufacturing); SYXK Gui 2017-0004 (Use)]. The raising conditions complied with the standards. The experiment was approved by the Ethics Committee of Youjiang Medical College for Nationalities. All rats were anesthetized by injected intraperitoneally with pentobarbital in all surgeries to reduce pain to the minimum.

### Reagents

Streptozotocin (STZ) was used in the study.

### Establishment and grouping of the animal model

Eighty healthy male Wistar rats were randomly assigned to control (*n =* 20) and diabetic ulcer wound groups (*n =* 60). The rats in the control group underwent full-thickness skin resection in the back (size: 2.5 × 2.5 cm^2^, with depth to the fascial layer) and were treated by applying physiological saline on the wound. The rats in the diabetic ulcer wound group were fed a high-fat diet for 4 weeks and then intraperitoneally injected with STZ (45 mg/kg) after being fasted for 16 h. The rat with fasting blood glucose ≥16.7 mmol/L was considered as a successful model of diabetes (Wang et al. 2018). After 1 week, an ulcer wound was made in the rat back (size and depth were the same as in the control group). *Staphylococcus aureus* suspension was smeared on the surface to make a diabetic ulcer wound infection model. These rats were further randomly assigned to MEBO, Kangfuxin and model groups (*n =* 20 in each group) and treated with MEBO (0.2 g/cm^2^), Kangfuxin liquid (0.2 g/cm^2^) and physiological saline, respectively.

### Specimen collection and processing

Rat wound healing was dynamically observed to calculate the healing rate. Wound healing rate = (original area − residual area)/original area × 100%. On days 1, 5, 11, 18 and 25, four rats in each group were randomly selected to be intraperitoneally injected with pentobarbital sodium for anesthesia (50 mL/kg). Wound tissues in the model area were collected to conduct pathological examination, biological examination and autophagosome observation by TEM.

### Histopathological assay

Wound tissues were fixed with 10% formalin for 48 h and stained with hematoxylin and eosin (H&E) after conventional embedding and sectioning. Histopathological changes were observed under an optical microscope.

### Observation of autophagosomes in ulcer wound tissue using a transmission electron microscope

The wound tissue specimens were fixed with the glutaraldehyde solution of transmission electron microscopy (TEM) grade, washed with phosphate-buffered saline, dehydrated and embedded with resin. Finally, a laboratory technician in the TEM lab conducted modification of the block, staining and image collection. The morphology and structure of autophagosomes in each specimen were observed under the same magnification.

### mRNA expression of PI3K, Akt and mTOR in rat ulcer wound tissues detected by qRT-PCR

Isolation of Total RNA and Real-time PCR Total RNA was isolated from the wound tissue of rats using TRIZOL according to the manufacturer’s instruction. Primers were designed first (Table 1). RNA was reverse transcribed to cDNA with Fast Quant RT kit. Real-time PCR analysis was performed using the SYBR Green. PCR reactions were performed using the following cycle conditions: pre-denaturation at 95 °C for 15 min, followed by denaturation at 95 °C for 10 s, annealing and extension at 60 °C for 20 s at the end, with 40 cycles in total. The relative amounts of the target mRNA were expressed as 2^−△△Ct^ as follows

(1) $$\Delta \Delta C_{t}=\;{\left(C_{T\;\text{target}}-C_{t\;\beta -\text{actin}}\right)}_{t}–{\left(C_{t\;\text{target}}-C_{t\;\beta -\text{actin}}\right)}_{c}$$

where *t* is the experimental group (MEBO, Kangfuxin and model groups), and *c* is the control group.

**Table 1.** Primer sequence and length.

Name	Sequence	Length
PI3K	F: 5′-TTCTGGTTCTTGCGAAGTGAGATAGC-3′	127bp
	R: 5′- GCGTGAAGTCCTGTAGCATGGC-3′	
Akt	F: 5′-CTCAACAACTTCTCAGTGGCACAATG-3′	82bp
	R: 5′- CAGGCAGCGGATGATGAAGGTG-3′	
mTOR	F: 5′-CTCAACAACTTCTCAGTGGCACAATG-3′	97bp
	R: 5′-CAGGCAGCGGATGATGAAGGTG-3′	

### Protein expression of PI3K, Akt and mTOR in rat ulcer wound tissues detected by Western blot analysis

The expression of PI3K, AKt, mTOR was measured by Western blot analysis. The wound tissue of each group was homogenized with an ultrasonic cell disrupter in 200 mL of ice-cold RIPA lysis buffer with protease inhibitors. Homogenates were centrifuged at 13,000 rpm at 4 °C for 10 min. Then supernatants were collected into a tube, and protein concentrations were determined using a BCA Protein Assay Kit. For Western blots, 40 μg protein was separated by 10% sodium dodecyl sulfate–polyacrylamide gels, subjected to electrophoresis and then transferred to PVDF (polyvinylidene difluoride) membranes. The immunoblot membranes were incubated with blocking solution containing 5% non-fat milk in TBS-T for 2 h at room temperature, followed by incubation overnight with primary antibodies on a shaker at 4 °C. Then, the primary antibodies were removed and the membranes were washed three times with Tris buffered saline–Tween 20 (TBS-T) and incubated with antirabbit horseradish peroxidase-conjugated IgG (1:50,000, ZSGB-BIO) for 2 h at room temperature. Subsequently, membranes were washed three times with TBS-T, then immunoreactivity was detected using an enhanced chemiluminescence reaction. The density of the bands was quantified by ImageJ version 1.41 (USA).1.2.7.

### Statistical analysis

The obtained data were analyzed using SPSS for Windows v17.0. Measurement data were expressed as mean ± standard deviation. Differences between multiple samples were analyzed by one-way analysis of variance, and pairwise comparison was tested by the least significant difference. A *p*-value <0.05 indicated a statistically significant difference.

## Results

### Establishment of a rat diabetic ulcer wound model

No change in food or water intake was observed in the control group, and body weight gradually increased with time. The rats in the ulcer wound groups showed increased food and water intake, excessive urination, emaciation, dry hair without luster, slow reaction and listlessness. As shown in Table 2, the blood glucose level in the three groups of diabetic ulcer wound on day 1 after modelling were all higher than 16.7 mmol/L, significantly higher than that in the control group (*F =* 940.35, *p <* 0.01). The rats in the MEBO, Kangfuxin and model groups were subcutaneously injected with insulin every day, and the blood glucose level was controlled within the range of 12–16 mmol/L, showing no statistically significant difference between the three groups (*F =* 0.235, *p =* 0.80).

**Table 2.** Comparison of blood glucose levels after modelling and during treatment in different groups.

Group	Blood glucose on day 1	Blood glucose on day 11
Control group	7.16 ± 0.39	7.30 ± 0.74
MEBO group	18.63 ± 1.00*	12.60 ± 1.62*
Kangfuxin group	18.53 ± 0.96*	13.04 ± 1.30*
Model group	18.10 ± 0.79*	12.90 ± 1.79*
*F*	940.35	45.61
*p*	<0.01	<0.01

*p < 0.01, compared with control group.

### Ulcer wound healing

**Figure 1.**
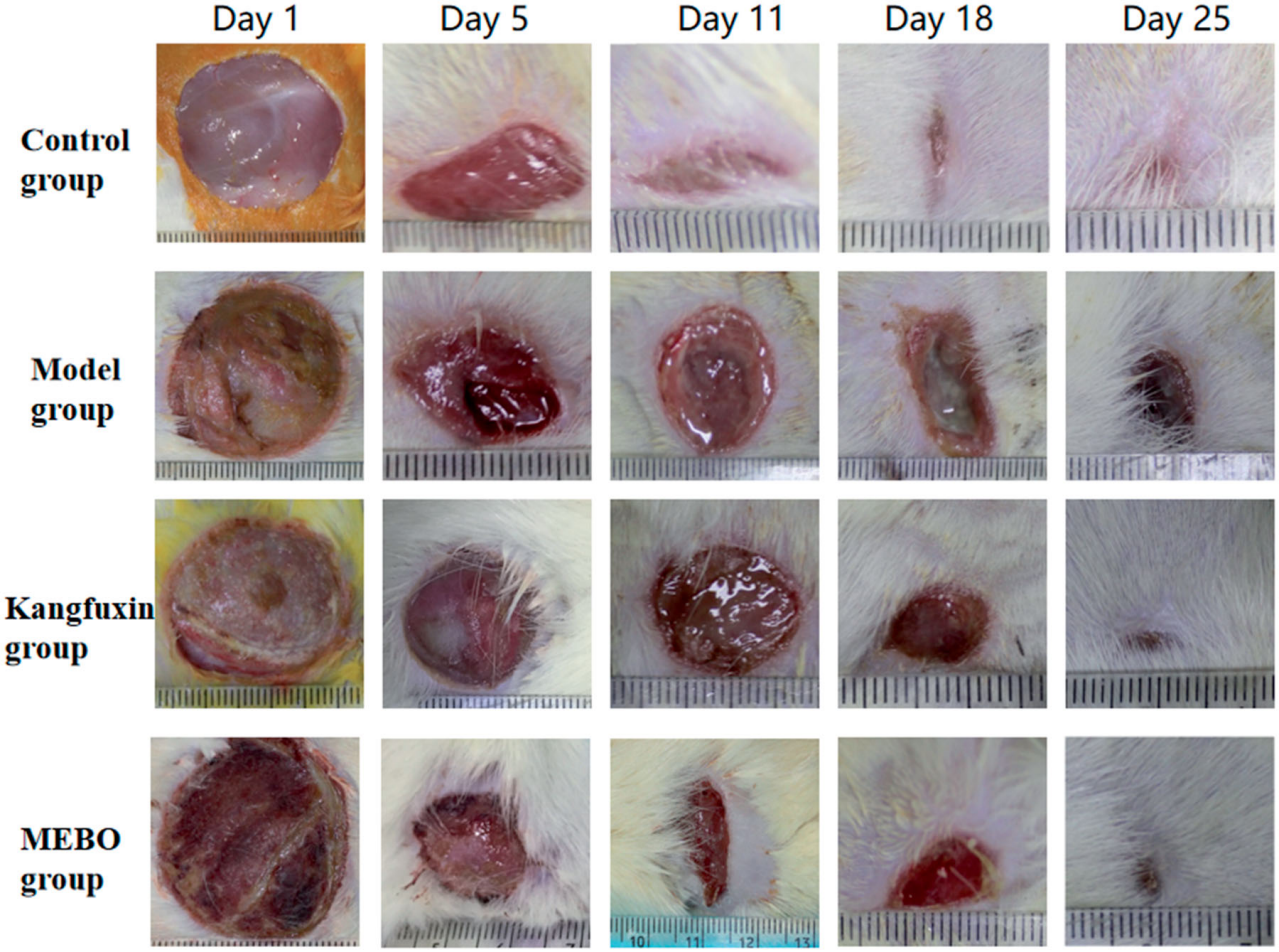
Comparison of healing of wounds in different groups at different time points.

Ulcer wounds could gradually heal over time in each group. The healing time in the control group was the shortest, and the wound almost healed on day 18. The condition in the model group was the worst, showing dark wound surface and red and swollen skin in the margin. On day 18, the exudation of purulent secretions was observed. The wound healing was relatively fast and exudation and suppuration were significantly alleviated in the MEBO group compared with the model group. On day 5, granulation tissue growth was obvious, and the wound almost healed on day 25. The healing condition in the Kangfuxin group was similar to that in the MEBO group, and the wound was almost healed (Figure 1).

#### Wound healing rate

The wound healing rate in each group gradually increased with time. On day 5, the wound healing rate was in the following order: control group > Kangfuxin group > MEBO group > model group (*p <* 0.05). On days 11, 18 and 25, the wound healing rate was in the following order: control group > MEBO group > Kangfuxin group > model group (*p <* 0.05). No statistically significant differences were found between the MEBO and Kangfuxin groups on days 18 and 25 (*p >* 0.05) (Table 3).

**Table 3.** Comparison of wound healing rates of rats in different groups at different time points (%).

Group	Day 5	Day 11	Day 18	Day 25
Control group	78.00 ± 0.00^▲^*	93.75 ± 5.00^▲^*	99.00 ± 0.00^▲^*	99.75 ± 5.00^▲^*
Model group	5.00 ± 8.16^△^*	60.25 ± 9.57^△^*	76.25 ± 2.22^△^*	89.00 ± 8.16^△^*
Kangfuxin group	67.00 ± 8.16^△▲^	76.25 ± 9.57^△▲^	93.00 ± 0.00^△▲^	98.00 ± 0.00^△▲^
MEBO group	61.25 ± 9.57^△▲^*	85.00 ± 8.16^△▲^*	93.25 ± 5.00^△▲^	98.50 ± 5.77^△▲^
*F*	10124.00	1187.00	298.39	311.13
*p*	<0.05	<0.05	<0.05	<0.05

At the same time point: ^△^*p <* 0.05, compared with the control group; ^▲^*p <* 0.05, compared with the model group; *p < 0.05, compared with Kangfuxin group.

### Histopathological results

**Figure 2.**
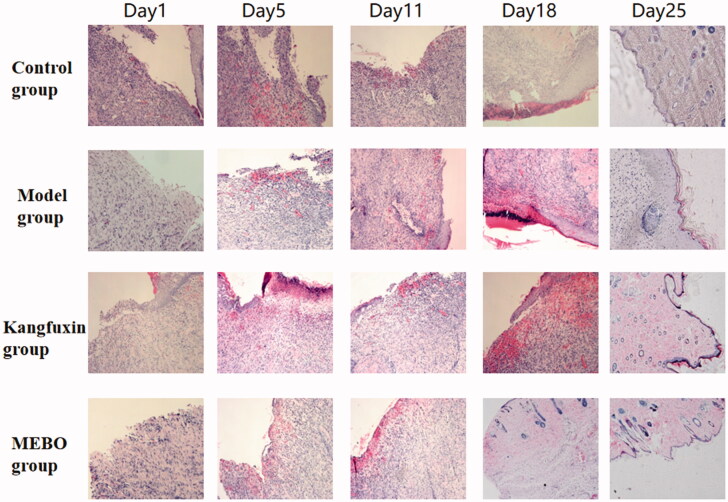
HE staining of wound tissue in different groups at different time points.

As illustrated in Figure 2, granulation tissue growth was observed on the ulcer wound surface, with the infiltration of inflammatory cells, under a microscope on days 5 and 11 after the treatment. The hemorrhage in the model was obvious with the exudation of more purulent liquid and some neovascularization. The inflammatory cell infiltration was less in the MEBO and Kangfuxin groups than in the model group; neovascularization was noted under the microscope. The rats in the control group completely healed on days 18 and 25 after the treatment. The necrotic tissues in the ulcer surface in the MEBO and Kangfuxin groups had immense neovascularization and dense distribution, forming a capillary network with an epidermal growth on the ulcer surface (Figure 2).

### Influence of MEBT/MEBO on autophagy in ulcer wound surface

**Figure 3.**
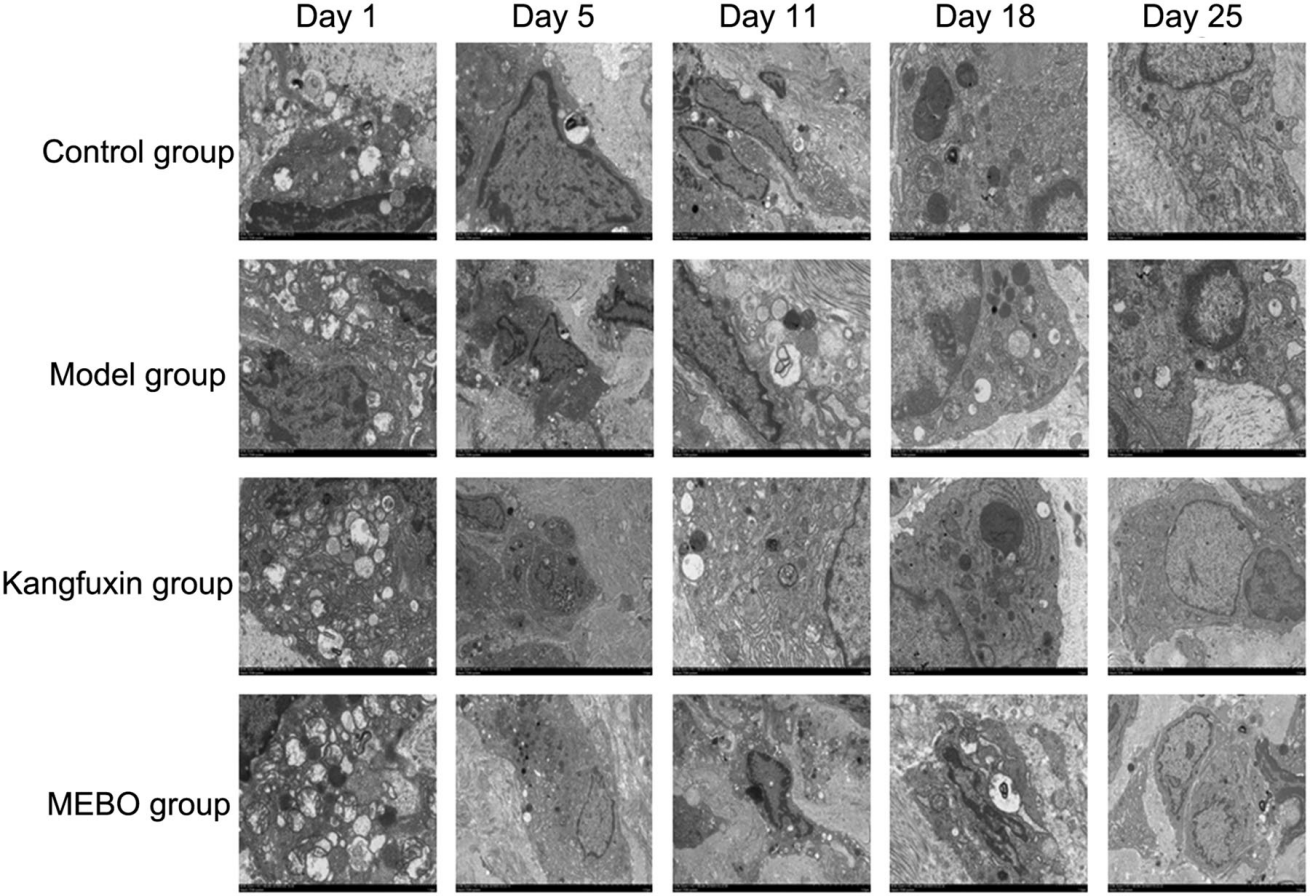
Autophagosome formation in wound tissue in different groups at different time points.

As illustrated in Figure 3, the morphology of autophagy in each group ranged from large to small. The autophagosomes were the fullest on day 1, showing a classic double-capsule structure. Dissolved residual organelles were observed, and some showed vacuoles after autophagic lysosome digestion. On day 5, autophagosomes became small or disappeared in each group; the change was the most obvious in the MEBO group. On day 11, 18 and 25, autophagosomes were relatively larger than those on day 5, but still significantly smaller than those on day 1.

### Influence of MEBT/MEBO on the expression levels of PI3K, Akt and mTOR mRNA in ulcer wounds

**Figure 4.**
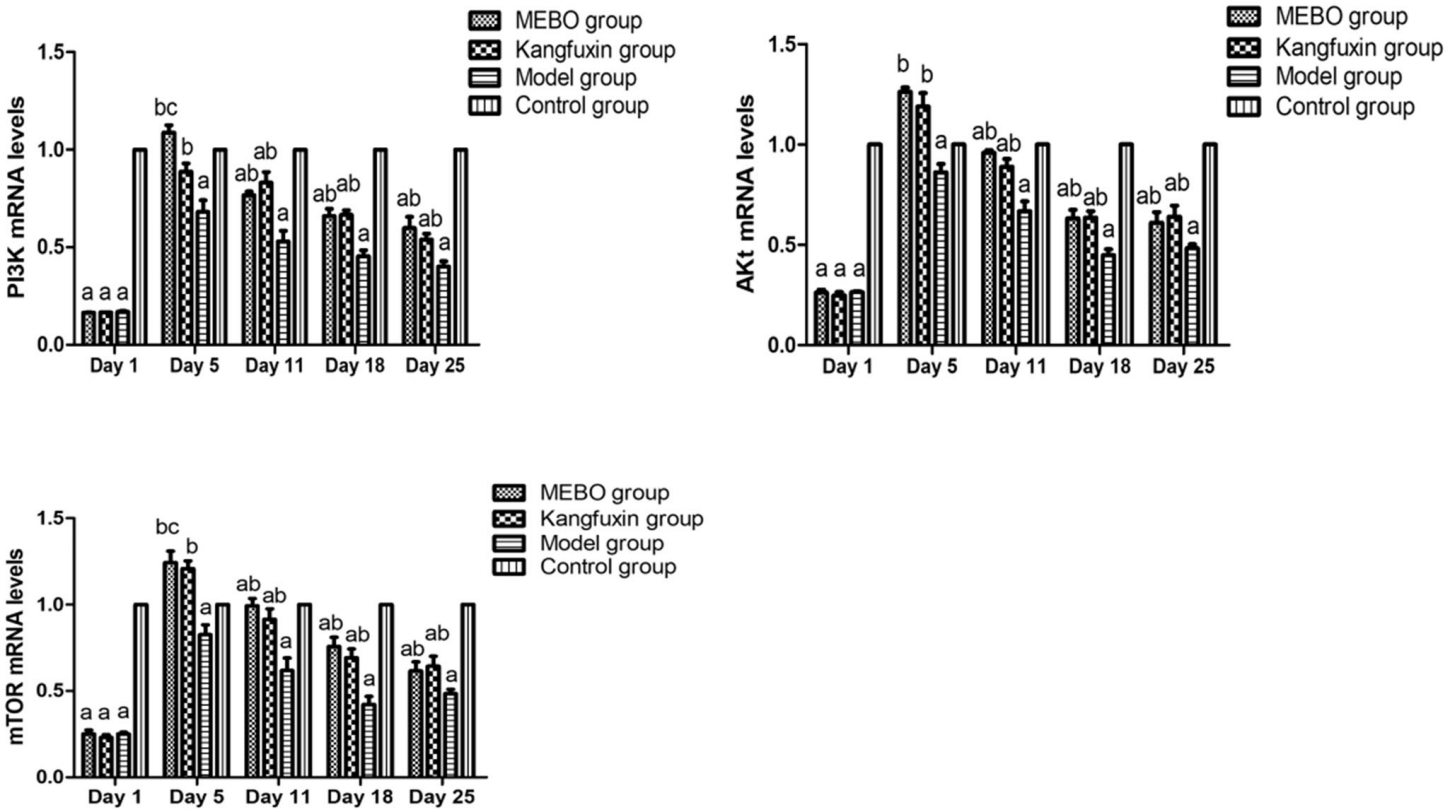
Expression of PI3K, Akt and mTOR mRNA in different groups at different time points. ^a^*p* < 0.05, compared with the control group; ^b^*p* < 0.05, compared with the model group; ^c^*p* < 0.05, compared with the Kangfuxin group.

(1) Comparison between groups at the same time point: On day 1, the expression levels of PI3K, Akt and mTOR mRNA in the diabetic ulcer wound groups (MEBO, Kangfuxin and model groups) was significantly reduced compared with the control group (*p <* 0.05), but without statistically significant difference between the three groups (*p >* 0.05). On day 5, the expression levels of PI3K, Akt and mTOR mRNA were the highest, with a statistically significant difference compared with the model group (*p <* 0.05). The expression in the MEBO group was the highest. The comparison between the Kangfuxin and model groups revealed statistically significant differences (*p <* 0.05), but no statistically significant difference compared with the control group (*p >* 0.05). On day 11, no statistically significant difference was observed between the MEBO and Kangfuxin groups (*p >* 0.05), but significant differences were found between the MEBO, and model and control groups (*p <* 0.05). The expression in the model group was still the lowest, with statistically significant differences compared with the other groups (*p <* 0.05). On days 18 and 25, the expression levels of PI3K, Akt and mTOR mRNA in each group continuously reduced, and the difference was the same as that on day 11. (2) Comparison within groups at different time points: The expression levels of PI3K, Akt and mTOR mRNA in each group of diabetic ulcer wound were the lowest on day 1 and reached the highest on day 5. The comparison at two time points showed statistically significant differences (*p <* 0.05). On day 11, the levels continuously reduced until days 18 and 25 (Figure 4).

### Influence of MEBT/MEBO on the expression levels of PI3K, Akt and mTOR proteins in ulcer wounds

**Figure 5.**
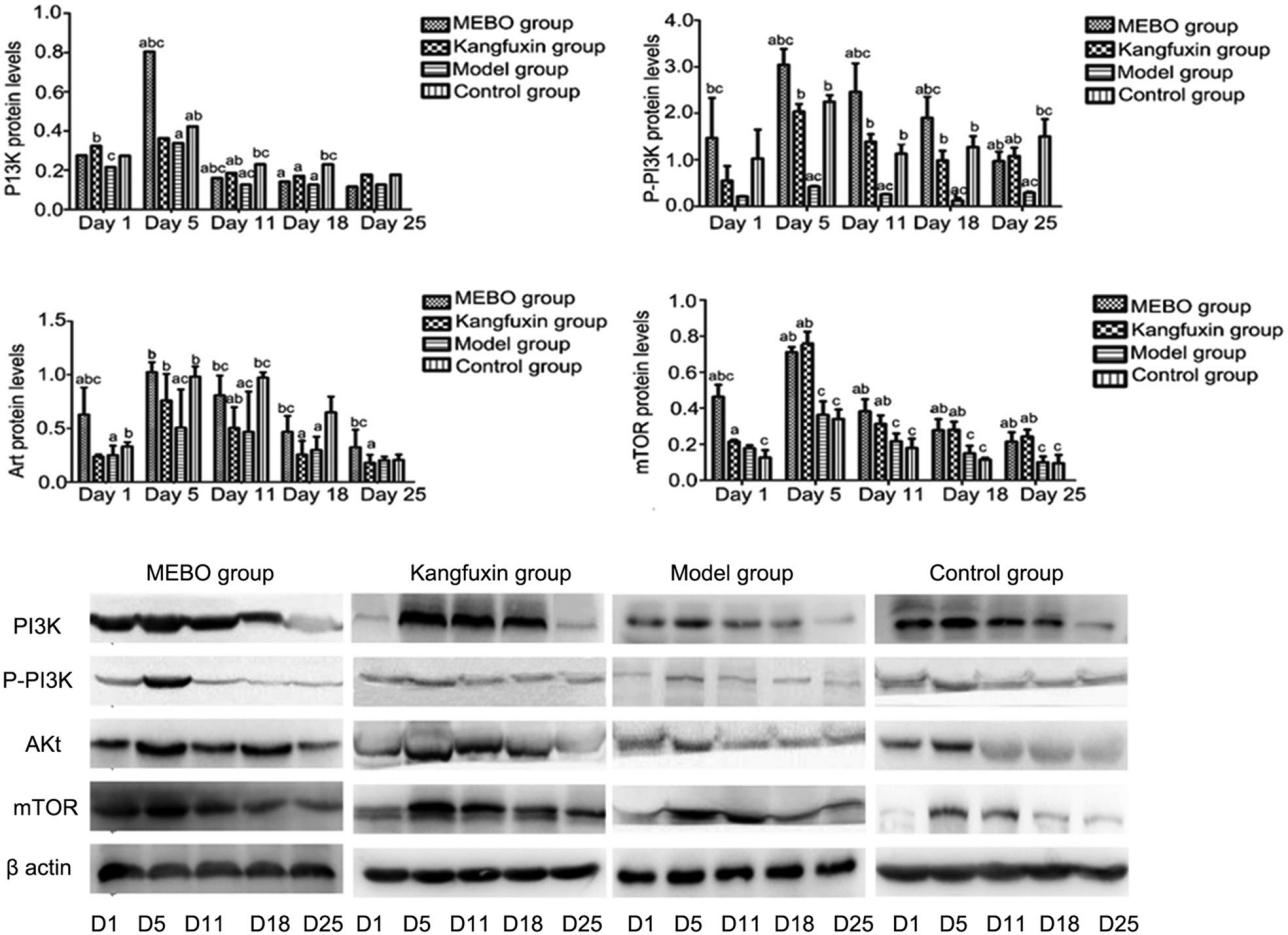
Expression of PI3K, P-PI3K, Akt and mTOR proteins on wound tissue in different groups at different time points ^a^*p* < 0.05, compared with the control group; ^b^*p* < 0.05, compared with the model group; ^c^*p* < 0.05, compared with the rehabilitation group.

(1) Comparison between groups at the same time point: At each time point, the expression levels of PI3K, P-PI3K, Akt and mTOR proteins in the model group were the lowest. On day 1, the expression levels of PI3K, P-PI3K, Akt and mTOR proteins in each group were significantly low. However, the MEBO group showed no statistically significant differences compared with the Kangfuxin, model, or control group (*p >* 0.05). On day 5, the expression levels in the MEBO group were the highest, with significant differences compared with the other three groups (*p <* 0.05). On day 11, the expression levels began to decline; the levels in the MEBO group were the highest, with statistically significant differences between the groups (*p <* 0.05). On day 18, the levels decreased in the following order: control group, Kangfuxin group, MEBO group and model group, with statistically significant differences between the control group and other groups (*p <* 0.05). However, the MEBO group showed no statistically significant differences compared with the Kangfuxin or model group (*p >* 0.05). On day 25, the expression levels continuously decreased, with no statistically significant differences in the pairwise comparison. (2) Comparison within groups at the same time point: The expression levels of PI3K, P-PI3K, Akt and mTOR proteins in each group were relatively low on day 1 and showed a peak on day 5 after the treatment. Then, the levels began to decline on day 11. The expression levels continuously decreased on days 18 and 25 of observation, with no statistically significant differences in the pairwise comparison on days 11, 18 and 25 (*p >* 0.05) (Figure 5).

## Discussion

The pathogenesis of diabetic ulcer wound is extremely complicated, involving vessels and neurologic impairment. The pathogenic process may be related to hyperglycemia, oxidative stress and inflammatory response (Patel et al. 2019). MEBT is an exposure therapy, MEBT/MEBO is a skin regenerative medicine and therapy, and MEBO is a TCM. Multiple previous investigations proved that MEBO could improve microcirculation, dissolve and excrete necrotic tissue, alleviate wound inflammation, improve vascular endothelial growth factor (VEGF) expression in wound surface, and promote wound tissue growth (Al-Meshaan et al. 2008; Tang et al. 2014; Fu et al. 2018; Hussein Ali and Adnan 2019; Zhu et al. 2019). The present study found that the healing time in the MEBO group was the shortest, and the wound healing rate at each time point was higher in the MEBO group compared with the model group. Exudation was less, and redness and swelling in the wound margin were not obvious. Under a microscope, revascularization in the MEBO group was found to be superior to that in the model group, and the capillary network was observed on day 5 with obvious granulation tissue growth. It indicated that MEBT/MEBO could alleviate wound inflammatory response, accelerate revascularization and formation of fibroblasts, and promote wound repair and healing.

Under TEM, autophagosomes in the wound in each group were from large to small, and the morphology in the early stage was the fullest with an obvious double-capsule structure. Then, it became smaller or disappeared in the middle and advanced stages, and the double-capsule structure was fuzzy. The change in the MEBO group was more obvious, suggesting that during autophagy the change was first enhanced and then weakened in the wound healing process. The regulatory effect of MEBT/MEBO on autophagosomes was superior to that in the other groups. Toll-like receptors are the first barrier against infection, which is related to autophagy (Levine et al. 2011; Into et al. 2012; Roedig et al. 2019). It influences immune response and inhibits inflammatory response via inducing autophagy. Revascularization is very important during wound healing. VEGF is the most specific growth factor in endothelial cells, which is very critical to vascularization (Fu et al. 2019). Many studies indicated that (Shen et al. 2013; Mondaca-Ruff et al. 2018) autophagy was related to common growth factors promoting vascularization, such as angiogenin and basic fibroblast growth factor. MEBT/MEBO promoted the repair of wounds and greatly improved wound repair quality in clinical applications without damaging the physiological environment of normal tissues.

The occurrence of cell autophagy needs the participation of many autophagy-related genes (Atg). For example, Unc-51 like autophagy activating kinase (ULK1)/Atg1 and Atg13 are essential for the occurrence of autophagy. The ULK1 complex is a formation bridge connecting upstream mTOR and downstream autophagosomes, which are critical positive regulators during autophagosome formation. Multiple signal transduction pathways participate in the regulation of autophagy. The PI3K–Akt–mTOR pathway is the most studied one and the only autophagy inhibitory pathway discovered so far (Kocaturk et al. 2019). mTOR senses multiple signals such as extracellular nutrition and oxidative stress, participates in intracellular signalling pathways, transfers extracellular nutrition signal with intracellular autophagy signal, and activates mTOR to inhibit autophagy (Guo et al. 2019). The present study found that the expression levels of critical factors (PI3K, Akt and mTOR) in the mTOR signalling pathway in rat ulcer wound tissue were relatively low, which increased later and then decreased. The trend in the MEBO group was more obvious. Combined with a previous study (Suvorova and Pospelov 2019), the present study deduced that in the early stage of the wound, the inflammatory response was evident, severe hypoxia and ischemia occurred in the wound, nutrition was lacking, energy was insufficient, adenosine triphosphate (ATP) level reduced, adenosine monophosphate (AMP)/ATP ratio increased, AMP-activated protein kinase (AMPK) was activated, and mTOR was inhibited. At this time, the inhibitory effect of mTOR on the phosphorylation of Atg1/ULK1 was attenuated, and the ULK1 complex was phosphorylated, inducing the occurrence of autophagy. Further, bacterial pathogens invading the wound were taken up by macrophages and transferred to intracellular lysosomes for degradation. Autophagy plays the roles of scavenging intracellular necrotic substances, resisting pathogens, regulating wound inflammatory response and promoting wound healing. Thus, in the early stage of the wound, autophagy is a kind of cell-protective mechanism. With stable control on the blood glucose level and extension of MEBO drug intervention time, MEBO could increase the synthesis and release of vascular endothelial cells and fibroblast growth factors, accelerate revascularization and improve blood flow on the wound surface. The abundant blood supply could transfer oxygen and nutrition to the wound, leading to the activation of mTOR sensing extracellular nutrient signalling. The activated mTOR binds to the ULK1 complex, resulting in the inactivation of ULK1 and inhibition of autophagy. It suggested that the reduced autophagy activation level might be the mechanism of MEBT/MEBO preventing epithelial hyperplasia and improving wound repair quality in the middle and advanced stages of the wound.

Also, at the same time point, the expression levels of PI3K, Akt and mTOR were persistently lower in the model group than in the control group, suggesting that the PI3K–Akt–mTOR signalling pathway was in a suppressed state in a diabetic ulcer wound. The possible reason might be related to hyperglycemia, oxidative stress and advanced glycation end products in the body. The comparison between the three groups of diabetic ulcer wound indicated that the expression levels of PI3K, Akt and mTOR were significantly higher in the MEBO group than in the model group, suggesting that MEBT/MEBO might activate the PI3K–Akt–mTOR signalling pathway and reverse the expression levels of PI3K, Akt and mTOR. Thus, MEBT/MEBO participates in ulcer wound repair and healing via regulating the PI3K–Akt–mTOR signalling pathway.
